# Epigenetic drugs induce the potency of classic chemotherapy, suppress post-treatment re-growth of breast cancer, but preserve the wound healing ability of stem cells

**DOI:** 10.1080/15384047.2022.2052540

**Published:** 2022-04-07

**Authors:** Andrew Zheng, Michelle Bilbao, Janhvi Sookram, Kimberly M. Linden, Andrew B. Morgan, Olga Ostrovsky

**Affiliations:** a Department of General Surgery, Cooper University Healthcare, Camden, NJ, USA; b Department of Gynecologic Oncology, MD Anderson Cancer Center at Cooper, Camden, NJ, USA; c Division of Surgical Research, Cooper University Healthcare, Camden, NJ, USA

**Keywords:** Triple negative breast cancer, epigenetic drugs, adipose-derived stem cells, wound healing, neoadjuvant chemotherapy, breast conserving therapy

## Abstract

Epigenetic therapy augments neoadjuvant chemotherapy (NACT) in breast cancer and may aid post-surgical wound healing affected by NACT. Our study investigates: (1) The cytotoxicity of classic paclitaxel chemotherapy on triple negative breast cancer (TNBC) independently and in combination with epigenetic drugs. (2) The sustainable inhibition of breast cancer regrowth following paclitaxel and epigenetic therapies. (3) The effects of paclitaxel with and without epigenetic therapy on the post-treatment viability and wound healing potential of adipose stem cells (ASCs). Cytotoxicity assays were performed on TNBC and ASCs. Cells were treated and recovered in drug-free medium. Cell viability was measured via cell counts and MTT assays. W -ound healing was tested with scratch assays. The combination of epigenetic drugs shows increased toxicity against TNBC cells compared to standard chemotherapy alone. Moreover, the combination of paclitaxel with epigenetic treatments causes cancer toxicity that is sustainable to TNBC cells after the drugs’ removal with minimal effect on ASCs wound healing ability. The use of epigenetic drugs in addition to standard chemotherapy is cytotoxic to TNBC cells and prevents post-treatment recovery of TNBC while maintaining ASC wound healing ability. This strategy may be useful in maximizing post-surgical wound healing following NACT in TNBC.

## Introduction

Breast cancer is the number one cause of cancer in women in the United States and the second cause of cancer death in this population.^1^ Of these, triple-negative breast cancer (TNBC) is the most aggressive molecular subtype.^2,3^ It lacks the three typical therapeutic targets: estrogen receptor (ER), progesterone receptor (PR), and human epidermal growth factor 2 receptor (HER-2). Despite having higher rates of pathologic complete response (pCR) than ER, PR, and HER-2 positive tumors, patients with TNBC still have lower rates of progression free and overall survival.^4^ The treatment of breast cancer involves a multidisciplinary approach consisting of chemotherapy, radiation, and surgical resection. Often, the first two modalities that are used are neoadjuvant chemotherapy and surgery.^5^ Therefore, efficient targeting of breast cancer together with protected post-surgical wound healing abilities are two significant and distinct criteria in the search for successful breast cancer therapies.

Neoadjuvant chemotherapy (NACT) is administered to patients with locally advanced breast cancer, select cases of early-stage breast cancer, patients with limited clinically node-positive disease, and patients with temporary contraindications for surgery. There are three goals to neoadjuvant chemotherapy. One is shrinking the tumor and decreasing the need for extensive breast and axillary resection. Additionally, NACT is associated with long-term disease-free survival and can elicit information about an individual patient’s tumor response.^6^ Finally, tumor response following neoadjuvant chemotherapy is especially important for patients with TNBC because patients with residual disease following NACT and surgery have a particularly poor prognosis.^4^

Taxanes, like paclitaxel, are typically used in the neoadjuvant setting for TNBC.^2^ While classic chemotherapy, like paclitaxel, has been essential in treating TNBC, its toxicities, including anemia, hair loss, arthralgias, myalgias, and peripheral neuropathy, limit its dosing and its cytotoxic potential.^7^ Furthermore, chemotherapy has the unfortunate side effect of impairing post-operative wound healing.^8^ Therefore, formulating treatment strategies that maximize tumor destruction while minimizing damage to healthy tissues is critical for good postoperative oncologic and esthetic outcomes.^9^

Paclitaxel has been shown to inhibit the proliferation and functional ability of local tissue stem cells critical for wound healing.^10,11^ Adipose-derived stem cells (ASCs) are pluripotent cells found in adipose tissue that play a critical role in postoperative wound healing. They have been extensively studied as an adjunct to breast reconstruction as well as improving the healing of chronic wounds including diabetic ulcers and limb ischemia.^11–15^ ASCs can be isolated from adipose tissue and their therapeutic applications are already apparent in techniques such as fat grafting.^15^ ASCs have paracrine effects on local cells and are capable of differentiating into several tissue types, including bone, cartilage, and adipose tissue.^16,17^ They play a major role in the regenerative microenvironment within the wound bed by inducing cell migration, differentiation, and angiogenesis.^18^ However, data are scarce regarding their resiliency to chemotherapeutic agents. Therefore, the development of more effective neoadjuvant therapy for TNBC is imperative, to not only achieve effective tumor destruction, but to preserve the survival and the ability of ASCs to support wound healing following surgery. To this clinical quandary, epigenetic therapy may be the solution.

Aberrant epigenetic modifications such as histone deacetylation and DNA demethylation can alter the expression of tumor suppressor genes or proto-oncogenes, leading to malignancy. There has been increasing interest in enhancing standard chemotherapy regimens by targeting these molecular mechanisms with epigenetic drugs.^19,20^ Epigenetic drugs can promote apoptosis and senescence and have been shown to be effective as adjuncts to chemotherapy regimens, in several *in vitro* and *in vivo* studies.^21–26^ Suberoylanilidehydroxamic acid (SAHA) is a histone deacetylase inhibitor (HDACi) that was approved by the Food and Drug Administration (FDA) for the treatment of cutaneous T-cell lymphoma and bladder cancer.^27,28^ Multiple recent studies have shown a beneficial effect in enhancing standard chemotherapy with HDACis in treating TNBC cells *in vitro*.^29–32^ Koko et al. demonstrated the additive effect of SAHA and paclitaxel in killing TNBCs in vitro, allowing the effective dosing of paclitaxel to be reduced.^33^ Although this method proved more effective compared to paclitaxel alone, the cancer cells remained incompletely eradicated and therefore, may potentially re-grow.

Azacitidine (AZA) is an FDA-approved DNA methyltransferase inhibitor (DNMTi) used in myelodysplastic syndrome, but small clinical trials have demonstrated some efficacy in treating solid tumors such as genitourinary and non-small cell lung cancer cells.^34–36^ Methylation and inactivation of breast cancer metastasis suppressor gene 1 (BRMS1) in TNBC cell lines and their role in the metastasis of breast cancer suggest that there may be a subgroup of patients who may potentially benefit from the use of DNMTi agents such as AZA.^37–39^

Therefore, we hypothesize that the addition of epigenetic therapy, AZA and SAHA, to standard chemotherapy, paclitaxel, will be more effective at reducing the preoperative tumor burden of TNBC while simultaneously preserving the wound healing ability of the surrounding ASCs. The first aim of our study was to evaluate the concurrent effect of AZA on TNBC and ASCs when combined with paclitaxel and SAHA. Our second goal was to investigate whether the addition of epigenetic drugs to paclitaxel could lead to sustainable TNBC cytotoxicity after drug removal. The final objective of our study was to examine the effects of paclitaxel with and without dual epigenetic therapy on the post-treatment viability and wound healing potential of ASCs.

## RESULTS

### IC_50_ determination

**Figure 1.**
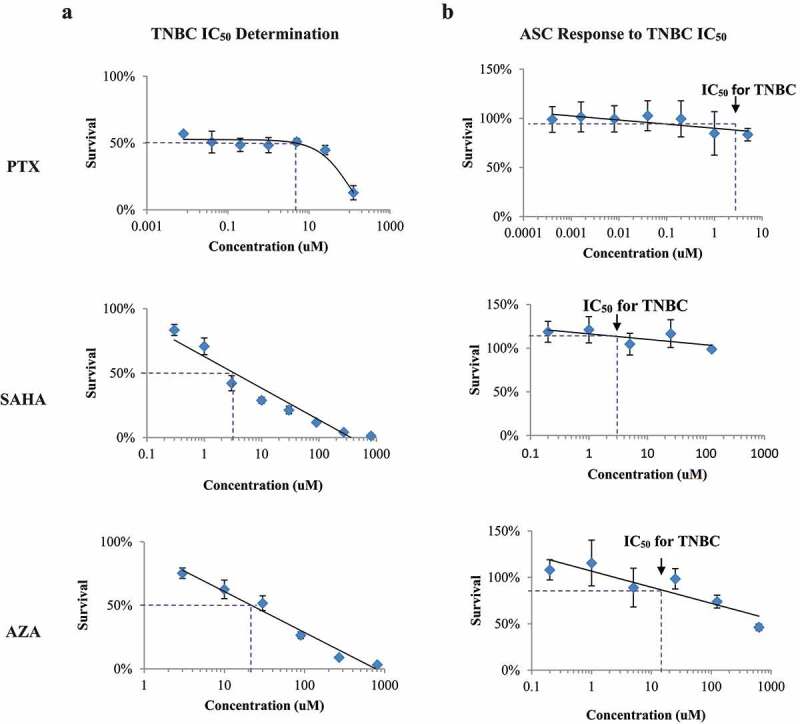
IC_50_ determination in TNBC (MDA-MB-231) and effect on ASCs. A. The TNBC cell line was exposed to serial dilutions of PTX, SAHA and AZA to determine the half maximal inhibitory concentration (IC_50_) of each drug. B. ASCs were also exposed to serial dilutions of PTX, SAHA and AZA. The IC_50_ of each drug in TNBC is noted on the survival curve of the ASC cells. (N = 4–7).

To use pharmacologically relevant drug concentrations, we determined the half-maximal inhibitory concentration (IC_50_) for each drug in TNBC cells. The IC_50_ concentrations for paclitaxel, SAHA and AZA in TNBC cells were determined to be 3.4 ± 2.0 µM, 3.11 ± 0.2 µM and 18.6 ± 3.6 µM respectively (Figure 1(a–c)). Interestingly, increasing doses of paclitaxel from 8 nM to 5 µM did not seem to affect TNBC cell survival (p = .306). The cells remained about 45–55% viable despite a greater than 1000-fold increase in paclitaxel concentration.

When the ASCs were treated with IC_50_ concentrations for TNBC, the same level of toxicity was not observed (Figure 1(d–f)). The TNBC IC_50_ for paclitaxel (3.4 µM) resulted in an 87.7% survival of ACSs (Figure 1(d)). Treatment with 3.1 µM SAHA resulted in 119% survival (Figure 1(e)) and treatment with 18.6 µM AZA resulted in 84.8% survival (Figure 1(f)). This demonstrated that the concentration of each drug that killed 50% of TNBCs had a minimal effect on ASC viability. These results are summarized in Table 1.

**Table 1.** ASC viability at TNBC IC50 treatments

Drug	Viability
PTX	87.7%
SAHA	119%
AZA	84.8%

### Combination treatment of paclitaxel with epigenetic therapy

**Figure 2.**
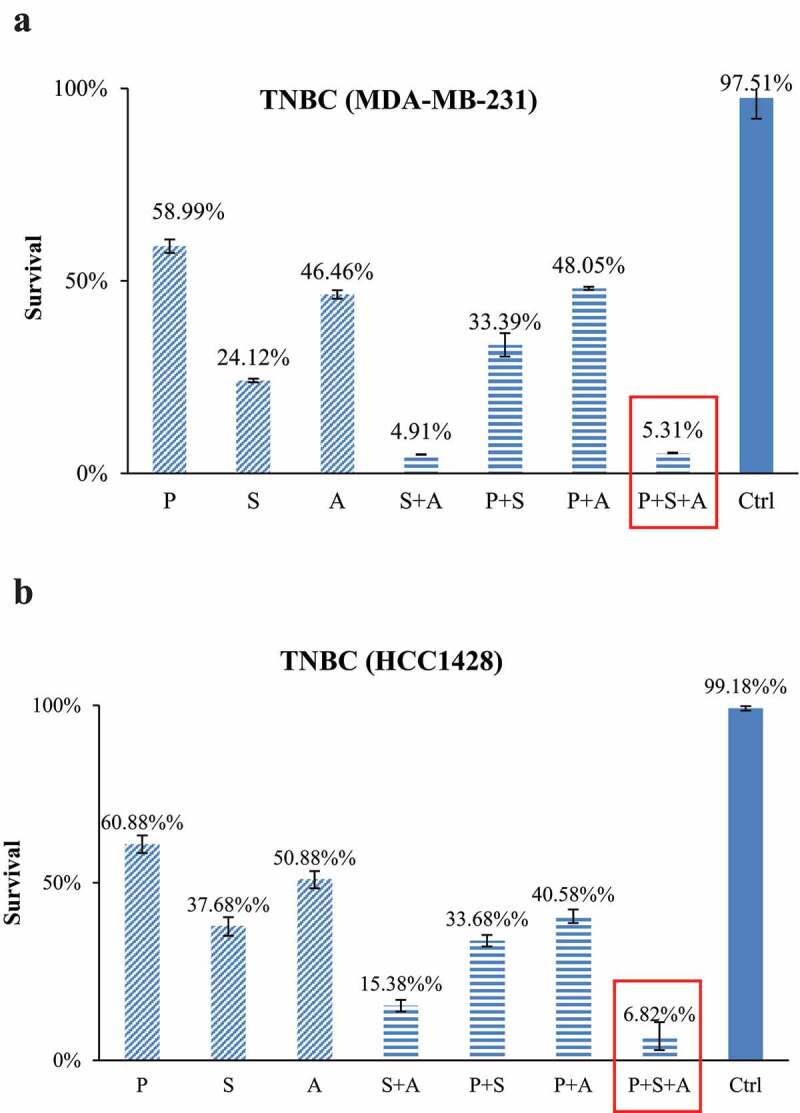
TNBC subjected to IC_50_ of PTX (p) alone or with combinations of IC50 of SAHA (s) and/or AZA (a). In the cell line MDA-MB-231, the combination of PTX with epigenetic drugs treatments significantly increased cytotoxicity compared to PTX alone (P < .001). (b). The cell line HCC1428 follows the same trend as MDA-MB-231, with the combination of epigenetic therapy and paclitaxel superior to the control as well as paclitaxel alone (P < .001). (N = 3).

To examine the cytotoxic effects of paclitaxel as a single agent or in combination with epigenetic regimens, both TNBC cells and ASCs were treated with combinations of paclitaxel, SAHA and AZA at their determined IC_50_ in TNBC cells (Tables 2 and 3). All treatments were statistically significant when compared individually to both the control and paclitaxel. The addition of SAHA to paclitaxel increased the killing efficiency of the drug regimen in the cell-line MDA-MB-231 by 26% (P < .001) and 27% in the cell-line HCC1428 (P < .001) (Figure 2). The combination of paclitaxel with AZA increased cytotoxicity by 19% compared with paclitaxel alone in MDA-MB-231 (P = .001) and 20% in HCC1428 (P < .001). In MDA-MB-231, the combination of both SAHA and AZA dramatically increased cytotoxicity even further to 95%, which was comparable to the efficacy of the combination SAHA-AZA-paclitaxel treatment. In HCC1428, the combination of SAHA, AZA and paclitaxel increased cytotoxicity to 93%, while the combination of SAHA + AZA increased cytotoxicity to 85%. In the latter cell line, the difference in these two treatments was statistically significant (P < .001) (Figure 2(b)).

**Table 2.** IC50 in TNBCs

Drug	IC50
PTX	3.41 ± 2.0uM
SAHA	3.11 ± 0.2uM
AZA	18.62 ± 3.6uM

**Table 3.** Drug treatment cocktails

Treatment groups	Combination of Drugs
Standard treatment	PTX
Epigenetic	SAHA
Treatments	
Alone	AZA
	SAHA + AZA
Standard and Epigenetic Co-treatment	PTX + SAHA PTX + AZA PTX + SAHA + AZA
Control	Media + DMSO

**Figure 3.**
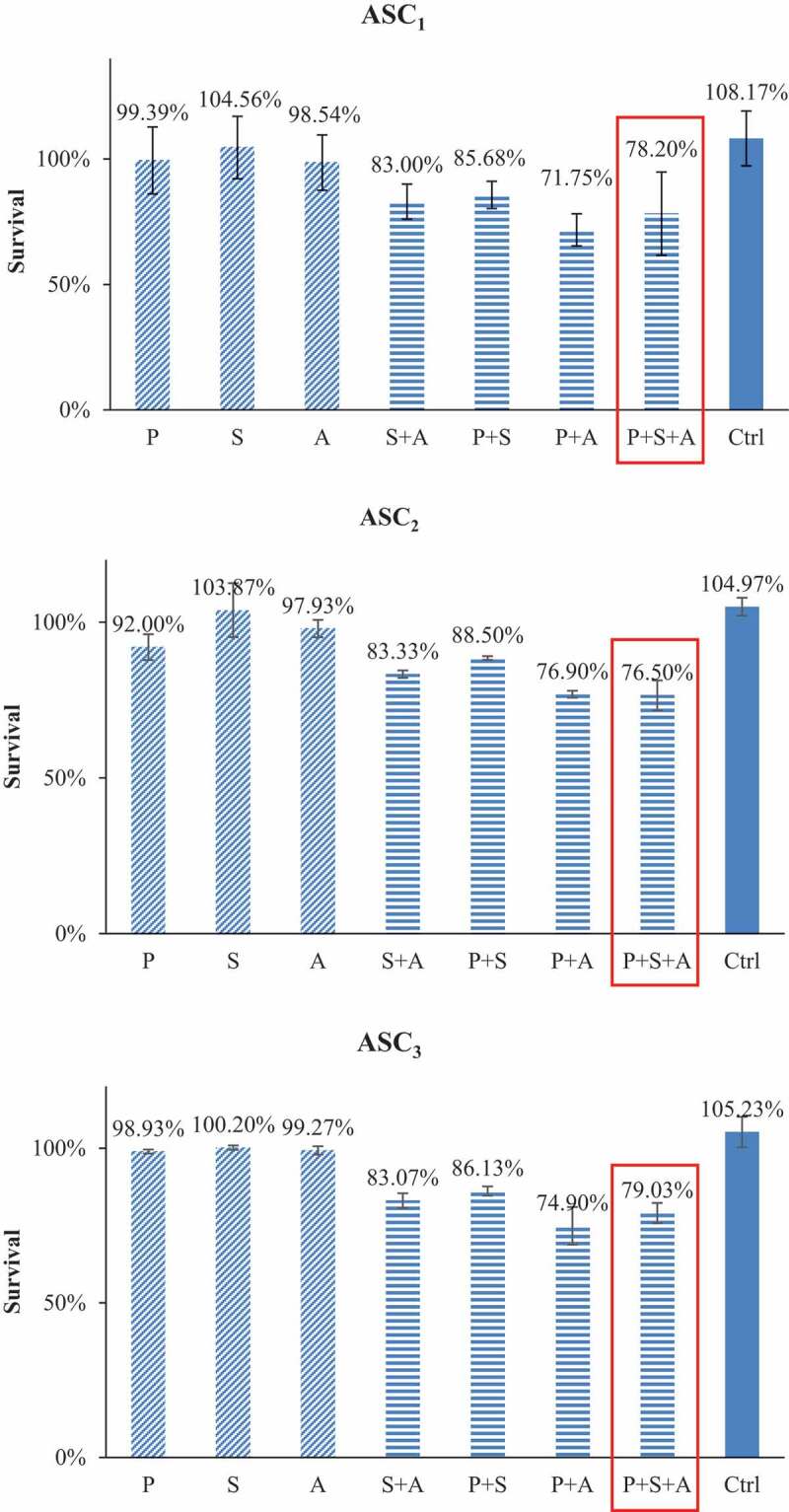
ASCs subjected to IC50 concentrations of TNBC cells for PTX alone or with combinations of IC50 concentrations of TNBC cells for SAHA or/and AZA. The combination of PTX with epigenetic drugs treatments had no difference in ASC survival versus PTX treatment alone. Cytotoxicity data from our three individual patients are shown (ASC_1_, ASC_2_, ASC_3_) (N = 3).

In the contrast, the same drug combinations did not cause statistically significant toxicity to ASCs (Figure 3). The addition of SAHA and AZA to paclitaxel only decreased ASC viability from 99% to 86% (P = .49) and 72% respectively (P = .56). The triple combination of SAHA-AZA-paclitaxel resulted in 78% viability of the ASCs (P = .16). After treatment with the triple combination, only 5% of the TNBC cells survived compared to 78% in the ASCs, a 73% difference in viability.

### Effects of paclitaxel with or without epigenetic drugs on TNBC and ASC cell survival

**Figure 4.**
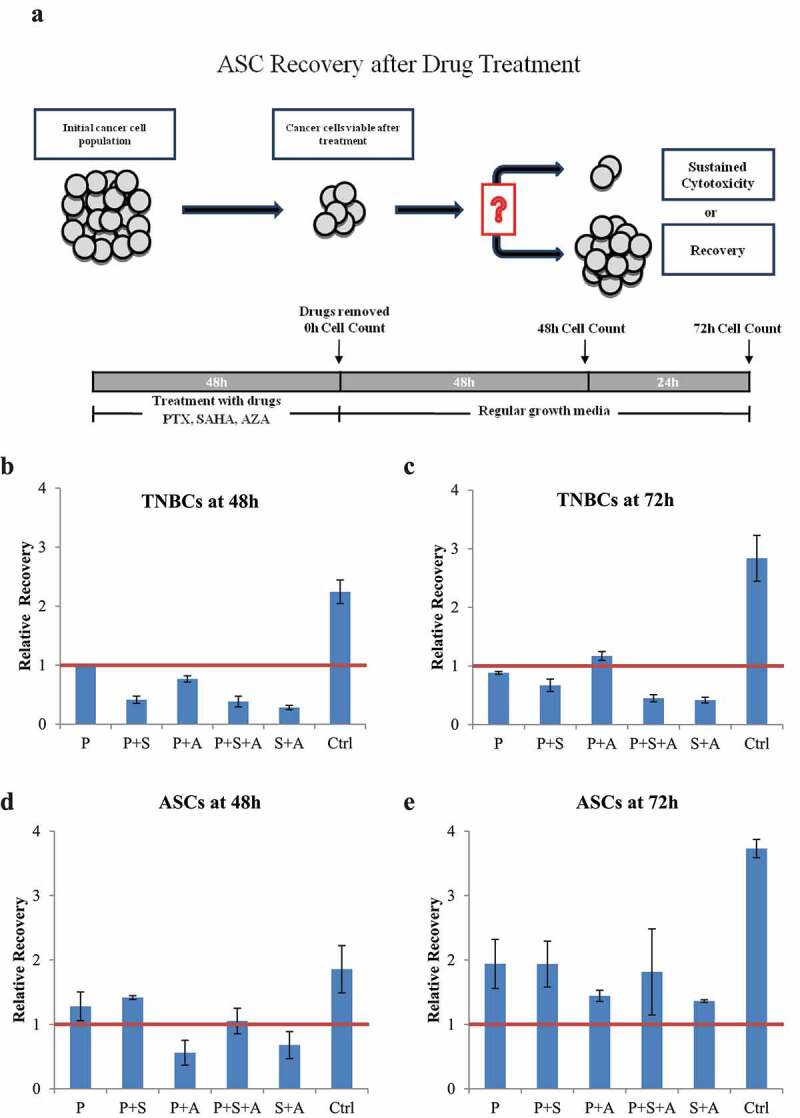
TNBC (MDA-MB-231) and ASC recovery after chemotherapy with and without epigenetic drug treatment (a). Schematic representation of experimental design of the drug recovery assay. (b, c). Recovery of TNBCs after 48 h (b) and after 72 h (c) after the drug removal, relative to post-treatment cell count. The PTX/SAHA/AZA treatment negatively affected recovery of TNBC cells even after drug removal and was significantly different from the control or PTX treatments alone at 48 h (P = .022 and p = .03, respectively). In a contrast, the post-treatment recovery of ASCs was not different between control or PTX treatment versus the PTX/SAHA/AZA treatment at 48 h D. (P = .15 and p = .39, receptively). ASCs show similar pattern, with no difference between control or PTX treatments versus PTX+SAHA+AZA at 72 h E. (P = .14 and p = .84, respectively). One-Way ANOVA and independent T test. (N = 3).

As seen in the schematic in Figure 4(a), we next analyzed paclitaxel and various epigenetic regimens on cancer cell regrowth after the drugs were removed from the culture. TNBC cells (MDA-MB-231) were treated with combinations of paclitaxel, SAHA and AZA. At 48 hours, the untreated TNBC control had proliferated twofold, while the paclitaxel treatment group cell count remained at the post-treatment cell count (Figure 4(b)). All other drug combinations resulted in continued cell death post-treatment. Interestingly, the potency of the epigenetic combination SAHA-AZA was very similar to the combination of epigenetic drugs with the classic chemotherapy, SAHA-AZA-paclitaxel. At 72 hours, the untreated control TNBC cells continued to proliferate, while the treated cells remained stagnant. Only the paclitaxel-AZA treated cells proliferated beyond the post-treatment number. The paclitaxel treated cells at this time displayed continued cell death.

With the same drug treatments, ASCs showed a different response pattern. Recovery after paclitaxel-AZA-SAHA cocktail did not show a significant difference with control treatment (P = .15 and P = .141) after 48- and 72-hour drug withdrawal, respectively. ASCs were able to proliferate beyond the initial post-treatment cell count after 72 hours of drug removal and have not shown a significant difference in the re-growth rate between the treatments (P = .464) (Figure 4(b)).

### Wound healing ability of ASCs with paclitaxel or combination of paclitaxel with epigenetic drugs

**Figure 5.**
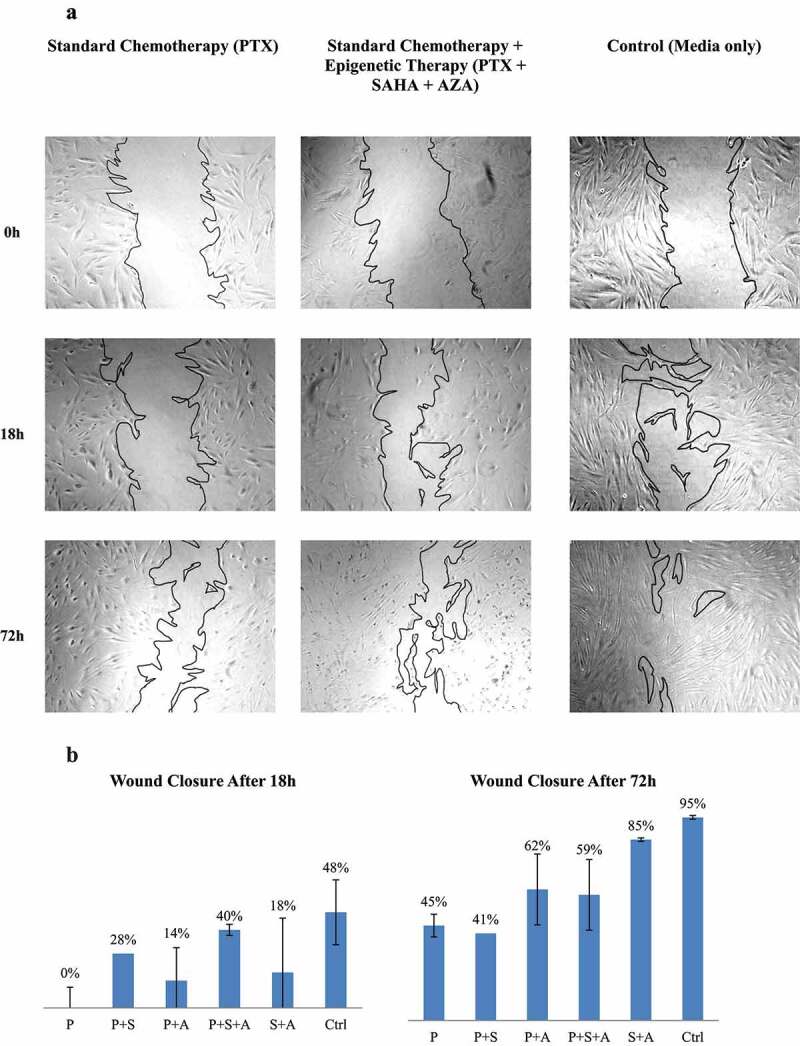
Effects of PTX and PTX with epigenetic treatments on wound closure in a scratch assay. A. Fibroblast scratch assays were incubated in conditioned media from ASCs (ASC_1_) treated with drugs. A. Wound closures for each treatment also are photographed at 5x magnification. B. Wound closure was measured after 18 h and 72 h. (N = 3).

Primary fibroblasts co-cultured with drug-treated ASCs showed an impaired ability to close a scratch “wound” assay. This was evident as non-treated ASCs consistently showed superior scratch closure recovery at each time point (Figure 5).

At 18 hours, the paclitaxel-treated ASC co-culture showed no healing of the scratch whatsoever. Interestingly, ASCs treated with combination treatments of SAHA and AZA with paclitaxel seemed to improve healing of the scratch. Despite being treated with all three drugs, ASCs that were treated with paclitaxel, SAHA and AZA still were able to heal 40% of the scratched wound at 18 hours and not much less than the 48% healed by the untreated ASCs (P = 1).

At 72 hours, primary fibroblasts co-cultured with untreated ASCs had closed nearly 100% of the scratch. Paclitaxel-only treated ASCs had healed 45% of the scratch, in contrast to 0% at 18 hours, but still exhibited a significant delay in wound healing as compared to control (P = .041). In contrast, the use of triple-drug therapy of paclitaxel with AZA and SAHA resulted in 59% of wound closure, with no significant statistical difference with the control treatment (P = .198).

In contrast to TNBCs, exposure of ASCs cells to the same epigenetic drug cocktails did not have any significant effect on ASCs viability or healing potential. Under the treatments, ASCs cells were able effectively proliferate and maintain their healing ability on wound closure of a primary fibroblasts scratch assay (Figures 2 and 5). Taken together, our data indicate that the addition of epigenetic drugs to paclitaxel not only enhanced cytotoxicity against of TNBC cells without extensive damage to ASCs, but also led to a sustained cytotoxic response in the cancer cells. This is significant as the upfront treatment of breast cancer relies not only on the ability of neoadjuvant chemotherapy to shrink the tumor, but also improved postoperative wound healing.

## Discussion

The management of TNBC is challenging due a lack of therapeutic targets and the aggressive nature of the disease. Standard neoadjuvant chemotherapeutic agents such as paclitaxel, while effective at killing tumor cells, have been shown to negatively impact ASCs’ viability and function, thereby having detrimental effects on wound healing and limiting its tumor killing potential.^40^ Optimal therapeutic regimens regarding TNBCs must account for not only the efficient targeting of breast cancer, but also for preserving wound healing after surgery.

Despite adequate initial treatment, a subset of cancer cells remains viable after treatment. This cancer cell population can regrow and metastasize, resulting in recurrent disease and cancer-related morbidity and mortality.^41,42^ Targeting cancer cells with different modalities and mechanisms, like epigenetic therapy, may be able to reduce their ability to re-grow following initial therapy. As evidenced, an additional benefit of epigenetic treatment is not only sustained cytotoxicity to cancer cells, but also the preservation of the viability and functionality of healthy tissues.

Epigenetic modifications in TNBC genes have been gaining tremendous interest as a target for therapies to halt breast cancer growth and metastasis.^37–39,43^ Clinical trials have shown that combinations of HDACis and paclitaxel have shown promising activity in TNBC.^44,45^ Our *in vitro* study has shown that the addition of the DNMTi AZA to this dual combination further potentiates their anti-proliferative effect in the TNBC cell line, MDA-MB-231 up to six-fold. The triple combination treatment of AZA-SAHA-paclitaxel caused 95% toxicity of TNBCs and only resulted in a mild decrease in ASC viability when compared to paclitaxel treatment alone. These findings are surprising, but can be explained in that the aberrant epigenetic modifications that are found in many breast cancer cells are reversed by our epigenetic interventions.^46^

The end result is cancer cell death with a minimal negative effect to the normal surrounding stroma. In fact, the viability and function of ASCs, cells that help drive local wound healing, were not significantly compromised (as compared to control of normal wound healing process) when exposed to the same concentration of this triple drug regimen. Based on these findings, we would like to note that the contrast between the negative effects of epigenetic drugs to cancer cells with the minimal adverse effects to normal cells may be used as an advantage in the development of the new effective strategies in breast cancer therapies. Indeed, other researchers in our lab have previously demonstrated that a combination of SAHA with paclitaxel increases paclitaxel-induced toxicity to TNBCs, allowing the effective dosing of paclitaxel to be reduced, decreasing toxicity to normal cells.^33^

Another challenge of treating TNBC is the regrowth of resistant cancer cells after treatment, eventually leading to cancer recurrence.^41,42^ Encouragingly, our recovery assay (Figure 4) demonstrated that the TNBC cell population remained inhibited after the drugs were removed from the media and even continued to die several days after that. Interesting, tumor cell death continued after cessation of all combinations of epigenetic treatments with paclitaxel except to the AZA-paclitaxel group. These data may reveal the fact that not all changes induced by epigenetic drugs result in cancer cell death. There is a high possibility that distinct tumor types and breast cancer at various stages may need different epigenetic drugs for the most efficient tumor eradication. Further studies are needed to elucidate the underlying molecular mechanism of this effects.

While paclitaxel, epigenetic therapy, and combinations of the two had maximal toxic effects on TNBC and minimal effects on ASC, it must be acknowledged that at this time our study does not provide the underlying molecular mechanisms for this effect. One possible explanation is that the existing aberrant epigenetic modifications found in TNBC are reprogrammed with epigenetic therapy, resulting in higher sensitivity to chemotherapy.^43^ Subsequent projects should investigate the regulation machinery and evaluate the changes in the molecular and biochemical processes in TNBC versus ASCs to discover more effective treatments for breast cancer patients. Another limitation of our study is that the recovery assays were performed over at 3-day duration. Future experiments should be followed in the long term (2–3 weeks) to understand more about the synergistic effect of epigenetic therapy with paclitaxel. Indeed, following these cells over a longer period of time may more closely reflect patient care outcomes, as patients are treated over several weeks and months, not several days. Moreover, this study has addressed the combination of epigenetic regimens with the standard therapeutic regimen paclitaxel only. Additional studies would need to be performed to demonstrate effects of epigenetic therapy with other chemotherapeutic drugs such as adriamycin and cyclophosphamide. Finally, this study has the inherent disadvantage of the *in vitro* design, a disadvantage that will necessitate verification in a future *in vivo* model.

Preclinical studies looking at combining chemotherapeutic treatments with two epigenetic agents with distinct pharmacologic approaches have been sparse. One recently completed phase II trial used a combination of epigenetic modulators such as AZA and entinostat (a class of HDACi) without cytotoxic chemotherapy in 13 women with TNBC. Although the combination was well-tolerated, there was no clinical response.^46^ Further clinical trials are needed to conclusively assess the clinical utility of HDAC inhibitors and DNMT inhibitors, specifically, their clinical responses, oncologic outcomes, and adverse pharmacologic effects in human patients.

In conclusion, the addition of AZA to a drug cocktail containing paclitaxel and SAHA to TNBC cells resulted in increased cytotoxicity to the TNBC cells without significant additional toxicity to ASCs. Moreover, the TNBC cell population remained inhibited after treatment and did not recover while ASCs retained their wound healing ability and continued to proliferate despite the treatment. Therefore, epigenetic therapy can be a useful adjunct to standard chemotherapeutic treatments to improve the toxicity to triple-negative breast cancer without significant damage to stem cells critical for healing post-surgical wounds.

## Materials & methods

### ASC isolation

Adipose tissue was collected from three volunteer patients during elective breast reduction surgery. In Figure 3, the cells from these patients are individually identified as ASC_1_, ASC_2_ and ASC_3_. Informed consent was obtained from the patient through the protocol approved by the institutional review board of Cooper University Hospital and Cooper Medical School of Rowan University. None of the patients had a history of malignancy, hence had not received neoadjuvant chemotherapy or radiation. The tissue was weighed and then rinsed with 1X phosphate buffered saline (PBS, Sigma) and incubated in a solution of with 1 mg/ml collagenase (Worthington) in 200 mg BSA (Gemini, West Sacramento, CA), resuspended in 1X PBS at a ratio of 1 milliliter collagenase solution per 1 gram of tissue, and then incubated for 1 hour at 37°C with vigorous shaking. Ten milliliters of M199 media (Cellgro, Manassas, VA) with 10% fetal bovine serum (Gemini, West Sacramento, CA) were added to inactivate the collagenase, and the resulting cell suspension was centrifuged at 1500 RPM for 10 minutes. The supernatant fluid and connective tissue were decanted, and the remaining cell pellet was resuspended in 10 mL of M199, filtered through a 100uM filter and centrifuged at 1200 RPM for 5 minutes. The supernatant fluid was aspirated and the pellet was briefly resuspended in 1 mL sterile distilled water to lyse the red blood cells followed by washing the pellet with 10 mL of M199 and centrifugation for a third time to obtain the stromal vascular fraction (SVF). An initial cell count of the SVF was determined using a Nucleotec NC-200 cell counter. The SVF cells were plated at 1 × 10^6^ per T75 flask and incubated overnight at 37°C. The following day, the media was discarded and replaced with fresh M199 with 10% fetal bovine serum (FBS).

### Cell culture

ASCs were grown in M199 with 10% FBS. Two TNBC cell lines were utilized, MDA-MB-231 and HCC1428. Both MDA-MB-231 and HCC1428 (ATCC, Manassas, VA) were grown in RPMI (Gibco, Gaithersburg, MD) with 10% FBS. Media was changed every 3–4 days and cells were passaged when they reached 80–90% confluence. ASCs were expanded until passage 4–5, when they were used for the experiments. All TNBC cells were at passage 8–10 when used for experiments.

### Drugs

Paclitaxel, suberanilohydroxamic acid (SAHA) and azacytidine (AZA) were purchased from Sigma-Aldrich and stored at −20°C. Stock solutions of paclitaxel, SAHA, and AZA were prepared by resuspending the powdered drug in 100% Dimethyl sulfoxide (DMSO, Sigma-Aldrich) and stored at −20°C.

### IC_50_ determination

In order to determine the half maximal inhibitory concentration (IC_50_) of each drug in TNBC cells, dose-dependent cytotoxicity assays were performed. TNBC cells were plated into 96 well plates at 6000 cells/well in triplicates and incubated at 37°C overnight. Serial dilutions were made under sterile conditions for each of paclitaxel, SAHA and AZA in RPMI with 10% FBS. The media was aspirated from the plated cells and replaced with 200uL of drug-containing media, returned to the 37°C incubator and treated for 48 hours. A methylthiazol tetrazolium (MTT) assay (Vybrant^TM^ MTT Cell Proliferation Assay Kit, Invitrogen, Eugene, OR) was used to assess for cell viability and cytotoxicity of experimental drugs (paclitaxel, SAHA and AZA) on ASCs and TNBC cells. The media from each well was aspirated and replaced with 100uL fresh media and 10uL stock MTT solution, and the plate returned to the 37°C incubator for 4 hours. One hundred microliters of SDS-0.01 M HCL solution from the MTT kit was then mixed into each well and the plate returned to the 37°C incubator. The plates were then read with the SpectraMax M3 plate reader (Molecular Devices, Sunnyvale, CA) to obtain absorbance readings at 570 nm. The percent survival was determined by taking the ratio of the absorbance of the treated cells in relation to an untreated control. The relationship between the percent survival versus drug concentration was graphed and the IC_50_ for each drug was determined from the formula of the logarithmic line of best fit.

### Combination treatment

Media containing combinations of paclitaxel, SAHA and AZA were made using the IC50 concentrations determined from our experiments (Table 2). ASCs and TNBC cells were plated and then treated with the combination treatment according to the same conditions as previously described. Percent survival was determined with an MTT assay and compared to an untreated control. Assays were run in triplicate and percent survival was calculated using One-way ANOVA and independent t-test.

### Post-treatment drug-recovery assay

ASCs and TNBC cells were plated in three 12 well plates (30,000 cells/well) in duplicate and treated with a combination of paclitaxel, SAHA and AZA for 24 h in a 37°C incubator. After 24 hours of treatment, one plate was trypsinized with 500uL of 0.5% trypsin and counted with a coulter counter. The other two plates had the media containing drug treatments removed and replaced with M199 with 10% FBS. Those plates were trypsinized at 48 hours and 72 hours and counted in the same manner. The relative proliferation at each time was determined by dividing the cell count by the initial count after drug treatment (time 0).

### Co-culture wound healing scratch assay

Primary fibroblasts (ATCC, Manassas, VA) were grown in Dulbecco’s Modified Eagle Medium (DMEM, low glucose, Hyclone, Logan, UT) with 10% FBS. One hundred thousand cells were seeded each in 12 well plates and incubated overnight. The next day, using a sterile 200ul pipet tip, a scratch was made in the carpet of fibroblasts. The media was aspirated and the cells washed with sterile PBS. The cells were then incubated in 1.2 ml of sterile serum-free M199 in preparation for co-culturing.

As the flasks with cells from the first patient (ASC_1_) demonstrated the largest number of cells in the lowest number of passages, ASC_1_ was used for conditioned media. ASCs were plated at 30,000 cells/ml with a total of 5 ml (150,000 cells) in six T25 flasks and allowed to incubate overnight for cell attachment. The media in each flask was removed the next day and replaced with the corresponding paclitaxel and epigenetic cocktail treatments as described above for 24 hours. At the end of treatment, the cells were washed with PBS and then replaced in their original media formulation and returned to the 37°C incubator for 48 hours to allow cells to recover. Cells were then trypsinized and 100,000 cells were placed into Costar Transwell (Sigma Aldrich, St. Louis, MO) 12 mm inserts in 1.2 ml M199 in 10% FBS, in 12 well plates. The inserts incubated overnight at 37°C to allow for attachment. The next day, the inserts were washed with sterile PBS, then transferred to the previously prepared primary fibroblast plates, and bathed in serum-free M199.

Pictures were taken using a Zeiss Axiovert 40 C microscope and AxioVisionLE software, at time 0 immediately after the scratch was created in the primary fibroblast carpet, as well as at 18, 36 and 72 hours after the scratch. Using forceps disinfected with 70% ethanol, the inserts were transferred to the empty 6 wells in the same plates, to allow for an unobstructed image to be taken. The inserts were replaced immediately after images were obtained and the plates returned to the 37°C incubator. The open scratch area was determined using ImageJ and numerical data analyzed using Microsoft Excel.

### Statistical analysis

All experiments were repeated three to seven times. The number (n) of repeated experiments is listed in the figure legends. Statistical analyses of viability were taken with independent t-tests and one-way analysis of variance (ANOVA) testing. P < .05 was used for statistical significance. Data analysis was performed using SPSS Statistics software version 22 (IBM, Armonk, NY).
